# Genome-Wide Association Studies Using 3VmrMLM Model Provide New Insights into Branched-Chain Amino Acid Contents in Rice Grains

**DOI:** 10.3390/plants12162970

**Published:** 2023-08-17

**Authors:** Yao Sui, Yanru Che, Yue Zhong, Liqiang He

**Affiliations:** School of Tropical Agriculture and Forestry, School of Tropical Crops, Hainan University, Haikou 570228, China

**Keywords:** rice, branched-chain amino acid, quantitative trait nucleotide, QTN-by-environment interaction, QTN-by-QTN interaction, 3VmrMLM

## Abstract

Rice (*Oryza sativa* L.) is a globally important food source providing carbohydrates, amino acids, and dietary fiber for humans and livestock. The branched-chain amino acid (BCAA) level is a complex trait related to the nutrient quality of rice. However, the genetic mechanism underlying the BCAA (valine, leucine, and isoleucine) accumulation in rice grains remains largely unclear. In this study, the grain BCAA contents and 239,055 SNPs of a diverse panel containing 422 rice accessions were adopted to perform a genome-wide association study (GWAS) using a recently proposed 3VmrMLM model. A total of 357 BCAA-content-associated main-effect quantitative trait nucleotides (QTNs) were identified from 15 datasets (12 BCAA content datasets and 3 BLUP datasets of BCAA). Furthermore, the allelic variation of two novel candidate genes, *LOC_Os01g52530* and *LOC_Os06g15420*, responsible for the isoleucine (Ile) content alteration were identified. To reveal the genetic basis of the potential interactions between the gene and environmental factor, 53 QTN-by-environment interactions (QEIs) were detected using the 3VmrMLM model. The *LOC_Os03g24460*, *LOC_Os01g55590*, and *LOC_Os12g31820* were considered as the candidate genes potentially contributing to the valine (Val), leucine (Leu), and isoleucine (Ile) accumulations, respectively. Additionally, 10 QTN-by-QTN interactions (QQIs) were detected using the 3VmrMLM model, which were putative gene-by-gene interactions related to the Leu and Ile contents. Taken together, these findings suggest that the implementation of the 3VmrMLM model in a GWAS may provide new insights into the deeper understanding of BCAA accumulation in rice grains. The identified QTNs/QEIs/QQIs serve as potential targets for the genetic improvement of rice with high BCAA levels.

## 1. Introduction

Rice is one of the most important staple foods, feeding more than half of the global population. It provides carbohydrates, amino acids, dietary fiber, etc., for the nutritional supplementation to humans [1,2,3,4]. Of the nine essential amino acids (valine, leucine, isoleucine, phenylalanine, tryptophan, threonine, lysine, methionine, and histidine), valine (Val), leucine (Leu), and isoleucine (Ile) are known as branched-chain amino acids (BCAAs), which are synthesized in plants and microbes, but not in humans [5,6]. Several studies have reviewed the nutritional importance of BCAAs in human health, such as protein synthesis, insulin secretion, and aging [5,7,8,9,10]. Due to the insufficient amount of BCAAs in rice grains, increasing the BCAA levels to meet the nutritional demands for dietary supplementation is becoming an emerging goal for the genetic improvement of nutrient quality.

A genome-wide association study, as a powerful tool, is widely used in the genetic dissection of complex traits controlled by polygenes [11,12,13]. For statistical models and their corresponding power of a GWAS, the adoption of single-locus model, such as GEMMA and MLM, has been proven to control spurious associations, and it has advantages in the detection of an association locus with a large effect [14,15]. However, low power in the detection of marker-trait associations may be affected by the stringent threshold via the Bonferroni correction [16]. To address this issue, a series of multi-locus GWAS models have been proposed to detect the quantitative trait nucleotide/locus (QTN/QTL) with a large and small effect in an efficient and accurate manner, such as MLMM, FarmCPU, mrMLM, pLARmEB, FASTmrEMMA, pKWmEB, FASTmrMLM, and ISIS EM-BLASSO [17,18,19,20,21,22,23]. To further decipher the genotypic effects including additive and dominance effects, recently, a new GWAS model based on the framework of mrMLM, namely 3VmrMLM, was proposed to estimate the additive and dominance effects of three marker genotypes (AA, Aa, and aa) using the polygenic background of the genotypic effects. Moreover, the main-effect QTN, QTN-by-environment interaction (QEI), and QTN-by-QTN interaction (QQI) can be detected using the 3VmrMLM model for a deeper understanding of the genetic architecture of the complex and multi-omics traits [24].

With the advances in technology and the low cost of metabolomics assays, tremendous progress has been made using the metabolite-based genome-wide association study (mGWAS) approach, which has been widely applied in the elucidation of the genetic and biochemical bases of the metabolite of interest [25,26,27,28]. Using the mGWAS strategy, several loci controlling the content alteration of primary and secondary metabolites were identified in plants [26,29,30,31,32,33,34,35]. As a primary metabolite, the main-effect QTNs/QTLs and candidate genes associated with the BCAA contents in rice were identified in previous studies [31,32]. For instance, the function of the *OsAUX5* (which encodes the amino acid transporter) gene in the uptake of valine and leucine was proven in our previous study of neutral essential amino acid accumulation in rice grains [30]. Moreover, the natural variation in *OsbZIP18* contributing to the BCAA levels was also reported in rice leaf [29]. Even though the genetic dissection of BCAA accumulation has been partially achieved, the genetic architectures of the QEI and QQI related to the BCAA levels in rice grains are largely unknown.

To obtain new insight into the genetic basis underlying the BCAA accumulation in rice grains, a genome-wide association study (GWAS) was performed on a diverse panel of 422 rice accessions with 239,055 SNPs; the association panel contained 272 *indica* accessions and 150 *japonica* accessions. Using the recently proposed 3VmrMLM model, the main-effect quantitative trait nucleotides (QTNs), QTN-by-environment interactions (QEIs), and QTN-by-QTN interactions (QQIs) associated with the BCAA accumulation were identified in the BCAA content datasets (one BCAA with two years and two replicates per year). The objective of this study is to identify the candidate genes that control the BCAA levels and are potentially involved in the gene-by-environment and gene-by-gene interactions in rice grains.

## 2. Results

### 2.1. Phenotypic Variation and Correlation Analysis

**Figure 1:**
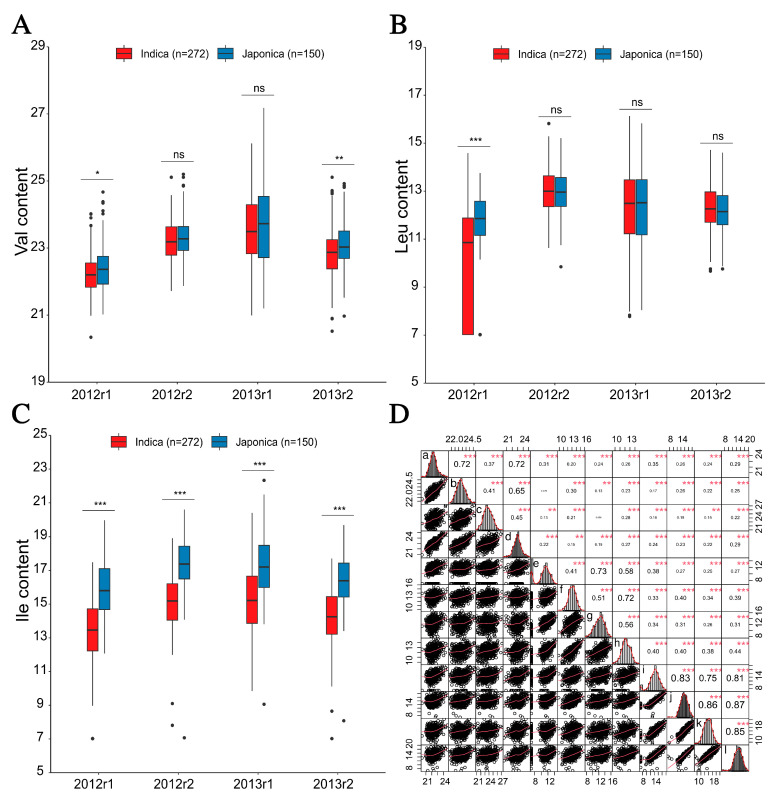
Grain branched-chain amino acid levels and correlation analyses in rice accessions. (**A**–**C**) Box plots of valine (Val), leucine (Leu), and isoleucine (Ile) contents for the 272 *indica* and 150 *japonica* accessions. 2012r1, 2012r2, 2013r1, and 2013r2 on the x axes represent Val, Leu, and Ile content datasets with two biological replicates in 2012 and 2013. The contents of Val, Leu, and Ile are shown on the y axes. (**D**) Distribution and correlation matrix of Val, Leu, and Ile contents with two replicates in 2012 and 2013. The letter from a to l indicate the first and second replicate in 2012 and 2013 of Val, Leu, and Ile contents, respectively. *, **, ***, and “ns” indicate statistical significance at the 5%, 1%, and 0.1% probability levels and no significant difference, respectively.

Using the LC-MS approach, the grain BCAA (Val for valine, Leu for leucine, and Ile for isoleucine) contents of 422 rice accessions were qualified for the analysis of phenotypic variation. As a result, a wide range of variation coefficient (CV) was observed from 53.65% to 219.03% across the 12 BCAA content datasets (Val, Leu, and Ile with two years by two replicates per year) (Table 1). Given that most of the skewness and kurtosis of the 12 content datasets were <1, these showed the quantitative nature of the BCAA contents. The estimated broad-sense heritability (*H*^2^) for Val, Leu, and Ile was from 0.45 to 0.82 (Table 1). Additionally, significant differences in the BCAA levels were observed between the *indica* and *japonica* accessions in this genetic panel (Figure 1A–C). For each BCAA, high correlation coefficients between the BCAA contents were observed across different content datasets. For instance, the significantly highest correlation coefficient with 0.87 was observed between the I_12r2 (the Ile content dataset of the second replicate of 2012) and I_13r2 datasets (the Ile content dataset of the second replicate of 2013) (Figure 1D). These indicated that the variation of the grain BCAA levels was present in this genetic panel.

### 2.2. Population Analysis of 422 Rice Accessions

**Figure 2:**
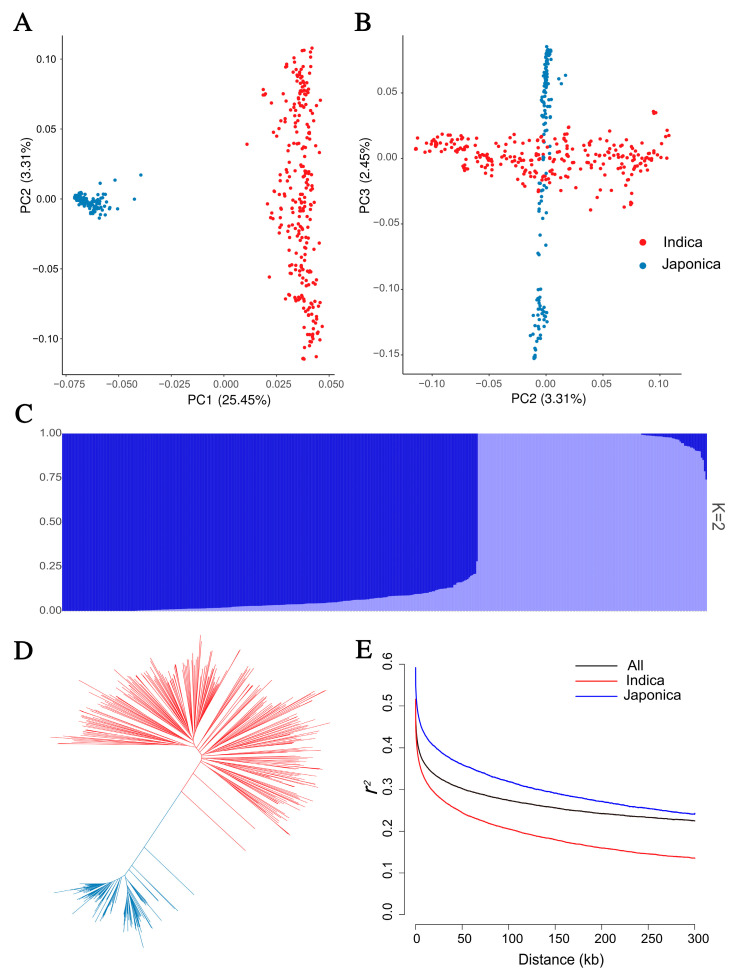
Population structure of 422 rice accessions. (**A**) and (**B**) are scatter plots of the first three principal components (PCs) of 422 rice accessions. The variation explained by the first and second PCs are shown on the x and y axes in (**A**). The variation explained by the second and third PCs are shown on the x and y axes in (**B**). (**C**) Population structure estimated via ADMIXTURE. (**D**) Phylogenetic analysis of 422 rice accessions, the *indica* and *japonica* accessions are indicated in red and blue. (**E**) Genome-wide LD decay analysis of the genetic panel; *indica* and *japonica* accessions are indicated in red and blue. The squared correlation coefficients (*r*^2^) between SNPs are shown on the y axis, and the distance of LD decay is shown on the x axis.

To dissect the genetic architecture of this rice panel, the relationship of 422 rice accessions were assessed on the basis of 239,055 SNPs. A principal component analysis (PCA) showed that these accessions were classified into two groups (Figure 2A,B), which mainly included 272 *indica* accessions and 150 *japonica* accessions. An ADIMIXTURE-based population structure analysis and neighbor-joining (NJ) tree-based phylogenetic analysis also showed identical results (Figure 2C,D). To investigate the linkage disequilibrium (LD) decay of this genetic panel, an *r*^2^-based LD analysis showed that the whole genome LD of all the accessions decayed the fastest before 122 kb, and became flat gradually (Figure 2E). In addition, the LD decay rate of the *indica* accessions was higher than the *japonica* accessions, which is also seen in Figure 2E.

### 2.3. BCAA-Level-Associated QTNs and Candidate Genes

**Figure 3:**
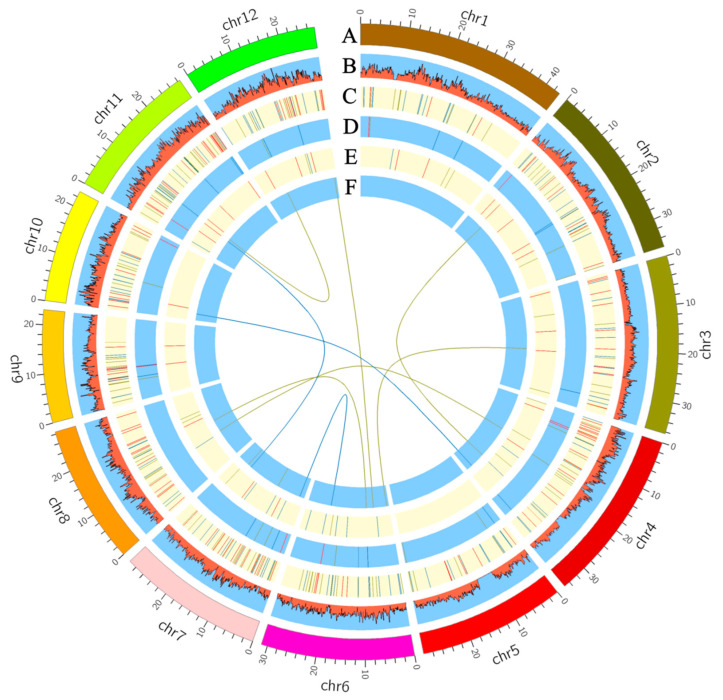
Circos map of quantitative trait nucleotides (QTNs), QTN-by-environment interactions (QEIs), and QTN-by-QTN interactions (QQIs) for branched-chain amino acid level in rice grains. Track (**A**): Twelve rice chromosomes. Track (**B**): Heatmap of SNP density with bin sizes of 0.1 Mb. Track (**C**): QTNs detected using 3VmrMLM model. Track (**D**): Common QTNs identified in two or more content datasets. Track (**E**,**F**): QEIs and QQIs detected using 3VmrMLM model. The red, green, and blue lines on the track C-F represent the QTNs/QEIs/QQIs associated with the grains’ Val, Leu, and Ile contents, respectively.

Using the newly released 3VmrMLM model, a total of 357 main-effect QTNs associated with the BCAA content were detected from 15 datasets (12 BCAA content datasets and 3 BLUP datasets of BCAA). The phenotypic variance explained (PVE) by each QTN ranged from 0.41% to 9.20%. Of these QTNs, 113, 125, and 125 QTNs were identified in the Val, Leu, and Ile content datasets, respectively (Figure 3 and Supplementary Table S1). Moreover, 41 common QTNs were detected in two or more content datasets, which contained 9 QTNs from the Val dataset, 13 QTNs from the Leu dataset, and 19 QTNs from the Ile dataset. The PVE of each common QTN was from 0.39% to 5.24% (Supplementary Table S2). Based on this, the allelic variation and BCAA content analyses were conducted to test the additive effect of these QTNs. The significant correlation coefficient between the number of favorable alleles (NFA) and the BCAA content was from 0.24 to 0.66. The highest correlation coefficient (*r* = 0.66) between the NFA and BCAA contents was observed in the Ile dataset (Ile_2012r1) (Supplementary Figure S3). For the candidate gene prediction, a total of 1537 genes were identified as putative genes underlying the BCAA accumulation (Supplementary Table S3). A KEGG pathway analysis showed that these genes were mainly involved in the biosynthesis of amino acids, valine, leucine, and isoleucine degradation and glutathione metabolism (Supplementary Figure S1A). Furthermore, allelic variations of *LOC_Os01g52530* and *LOC_Os06g15420* associated with the Ile content alteration were identified. The *LOC_Os01g52530* encoding phosphoribosyl transferase is localized in the LD block (Chr1: 30,117,297–30,187,297 bp) of the QTN-sf0130120297 (sf0130120297 represents chromosome 1 at 30,120,297 bp) locus (Supplementary Figure S2A and Supplementary Table S3). The *LOC_Os06g15420* encoding asparagine synthetase was in the LD block (Chr6: 8,755,681–8,775,681 bp) of the QTN-sf0608765681 locus (Supplementary Figure S2C and Supplementary Table S3). The accessions carrying functional allele2 (*n* = 215) of *LOC_Os01g52530* with the GG allele showed a significantly higher Ile level than the accessions carrying functional allele1 (AA, *n* = 207) (Supplementary Figure S2B). Likewise, the accessions carrying functional allele2 (*n* = 363) of *LOC_Os06g15420* with the TT allele showed a significantly higher Ile level than the accessions carrying functional allele1 (AA, *n* = 59) (Supplementary Figure S2D).

### 2.4. BCAA-Level-Related QEIs and Candidate Genes

**Figure 4:**
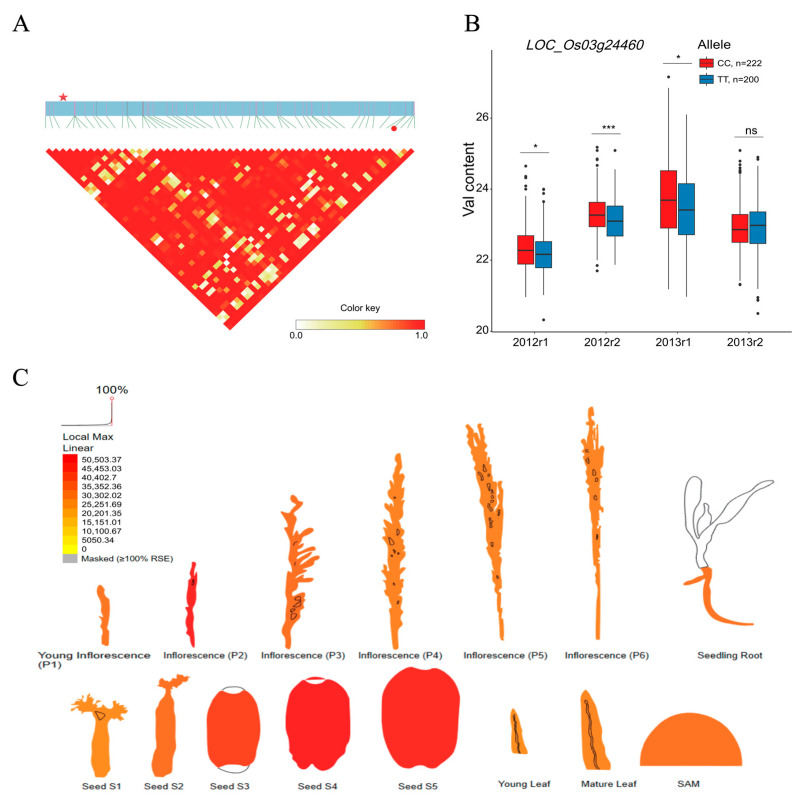
Analyses of the candidate gene *LOC_Os03g24460* related to valine (Val) content. (**A**) Local linkage disequilibrium block analysis for *LOC_Os03g24460* and QEI-sf0314026224 locus. (**B**) Allelic variation and Val content analysis of *LOC_Os03g24460* in 422 rice accessions with two replicates in 2012 and 2013. The Val content and content dataset info are shown on the y and x axes. 2012r1, 2012r2, 2013r1, and 2013r2 represent two replicates in 2012 and 2013. (**C**) Expression profile of *LOC_Os03g24460* based on ePlant transcriptome data in rice, with the expression strength coded by color from yellow (low) to red (high). Red star and red dot indicate *LOC_Os03g24460* and QEI-sf0314026224. *, ***, and “ns” indicate statistical significance at the 5% and 0.1% probability levels and no significant difference, respectively.

**Figure 5:**
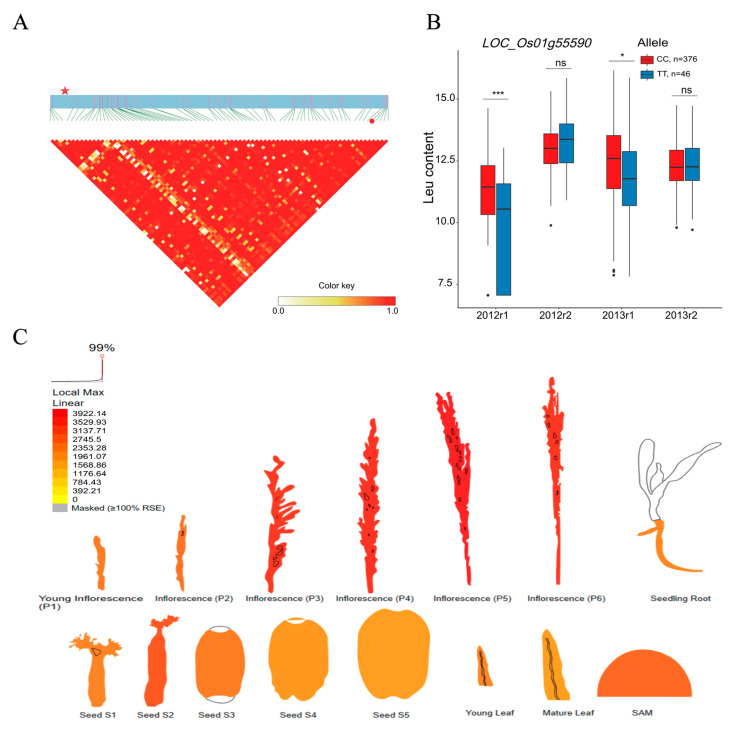
Analyses of the candidate gene *LOC_Os01g55590* related to leucine (Leu) content. (**A**) Local linkage disequilibrium block analysis for *LOC_Os01g55590* and QEI-sf0132146191 locus. (**B**) Allelic variation and Leu content analysis of *LOC_Os01g55590* in 422 rice accessions with two replicates in 2012 and 2013. The Leu content and content dataset info are shown on the y and x axes. 2012r1, 2012r2, 2013r1, and 2013r2 represent two replicates in 2012 and 2013. (**C**) Expression profile of *LOC_Os01g55590* based on ePlant transcriptome data in rice, with the expression strength coded by color from yellow (low) to red (high). Red star and red dot indicate *LOC_Os01g55590* and QEI-sf0132146191. *, ***, and “ns” indicate statistical significance at the 5% and 0.1% probability levels and no significant difference, respectively.

**Figure 6:**
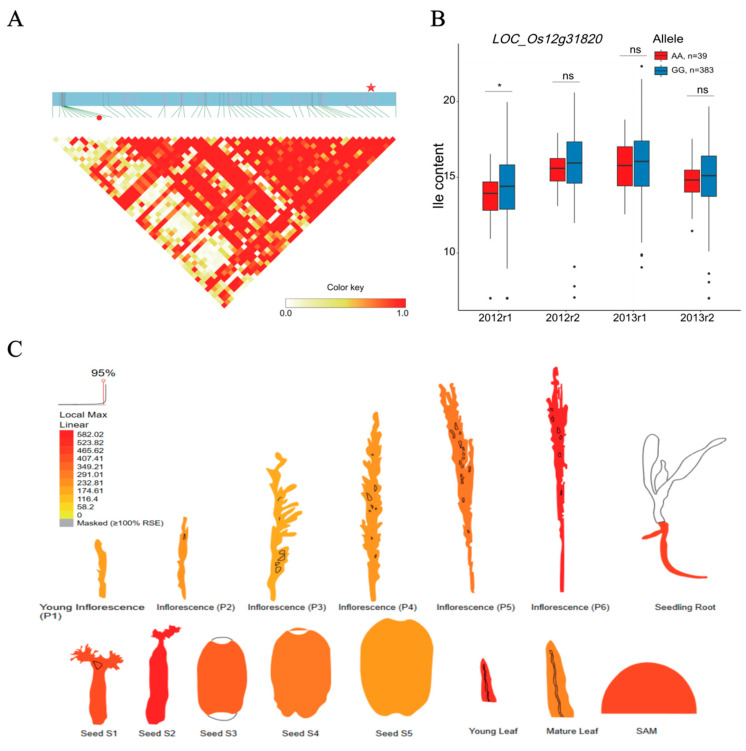
Analyses of the candidate gene *LOC_Os12g31820* related to isoleucine (Ile) content. (**A**) Local linkage disequilibrium block analysis for *LOC_Os12g31820* and QEI-sf1219051313 locus. (**B**) Allelic variation and Ile content analysis of *LOC_Os12g31820* in 422 rice accessions with two replicates in 2012 and 2013. The Ile content and content dataset info are shown on the y and x axes. 2012r1, 2012r2, 2013r1, and 2013r2 represent two replicates in 2012 and 2013. (**C**) Expression profile of *LOC_Os12g31820* based on ePlant transcriptome data in rice, with expression strength coded by color from yellow (low) to red (high). Red star and red dot indicate *LOC_Os12g31820* and QEI-sf1219051313. * and “ns” indicate statistical significance at the 5% probability level and no significant difference.

In total, 53 QEIs were detected in three BCAA content datasets, which accounted for the potential interactions between the gene and environmental factor (Figure 3 and Supplementary Figure S4). The numbers of QEIs related to the grains’ Val, Leu, and Ile accumulations were 26, 13, and 14, respectively. The PVE of each QEI ranged from 0.15% to 3.06%. No common QEI was found in the three BCAA content datasets (Figure 3 and Supplementary Figure S4). Interestingly, QEI-sf0227136932 was also identified as the main-effect QTN associated with the Leu accumulation (Supplementary Tables S1 and S4). To further elucidate the genetic mechanism responsible for the gene and environment interaction, a total of 1921 genes were identified as putative genes underlying the grain BCAA accumulation (Supplementary Table S5). According to the KEGG annotation, these genes gathered in valine, leucine, and isoleucine degradation, in the biosynthesis of amino acids, and in amino-acid-related enzymes (Supplementary Figure S1B). Moreover, several genes harbored in the QEI loci and potentially related to the variation of BCAA accumulation were identified. For instance, *LOC_Os03g24460* in the LD block (Chr3: 13,941,224–14,029,224 bp) of the QEI-sf0314026224 locus encoding aminotransferase-domain-containing protein was related to the content variation of the BCAA Val (Figure 4A and Supplementary Table S5), which presented two types of alleles, functional allele1 (CC, *n* = 222) and functional allele2 (TT, *n* = 200). A significantly higher Val content was observed in the accessions with allele1 than in the accessions with allele2 in the 2012r1, 2012r2, and 2013r1 datasets (Figure 4B). The ePlant gene expression profile showed that *LOC_Os03g24460* was highly expressed in seed (S5, S4, and S3) and inflorescence (P2) (Figure 4C). Additionally, *LOC_Os01g55590* in the LD block (Chr1: 32,024,191–32,149,191 bp) of the QEI-sf0132146191 locus encoding AMP-binding enzyme was identified as the key gene associated with the content alteration of the BCAA Leu (Figure 5A and Supplementary Table S5), which had two types of alleles, functional allele1 (CC, *n* = 376) and functional allele2 (TT, *n* = 46). The accessions carrying allele1 exhibited a significantly higher Leu level than those carrying allele2 in the 2012r1 and 2013r1 datasets (Figure 5B). *LOC_Os01g55590* was relatively highly expressed in inflorescence (P3, P4, P5, and P6) and seed (S2) based on the ePlant expression data (Figure 5C). Additionally, *LOC_Os12g31820* in the LD block (Chr12: 19,048,313–19,124,313 bp) of the QEI-sf1219051313 locus encoding phosphoserine phosphatase protein was identified as the candidate gene responsible for the content alteration of the BCAA Ile (Figure 6A and Supplementary Table S5), which presented two types of alleles, functional allele1 (AA, *n* = 39) and functional allele2 (GG, *n* = 383). The accessions carrying allele1 showed a significantly lower Ile content than those carrying allele2 in the 2012r1 dataset (Figure 6B). A high expression of *LOC_Os12g31820* was observed in seed (S2), inflorescence (P6), and young leaf based on the ePlant analysis (Figure 6C).

### 2.5. BCAA-Level-Related QQIs

**Figure 7:**
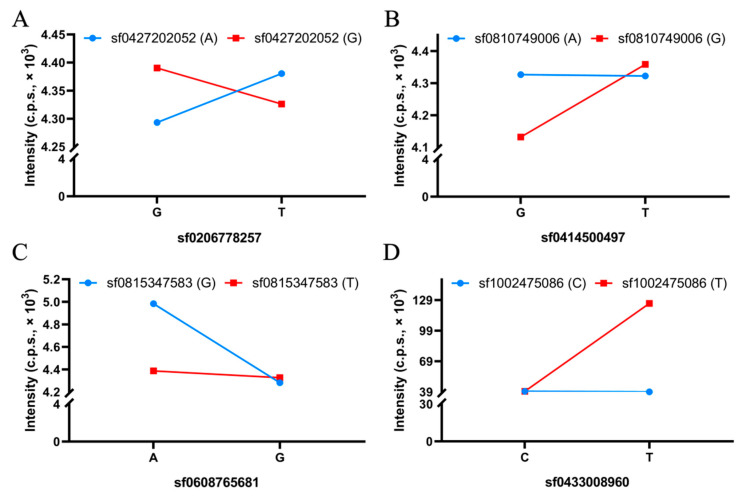
Detected QTN-by-QTN interactions (QQIs) related to branched-chain amino acid (BCAA) content in rice grains. (**A**–**C**) Interaction between two QTNs underlying the leucine (Leu) accumulation. (**D**) Interaction between two QTNs underlying the isoleucine (Ile) accumulation. The alleles of first QTN and intensity of the accessions carrying corresponding alleles are shown on the x and y axes of each plot. c.p.s. indicates counts per second.

QTN-by-QTN interaction (QQI) detection is an important and complementary approach for the full genetic view of the BCAA accumulation in rice grains. Using the 3VmrMLM model, a total of 10 QQIs were detected in two BCAA content datasets (Figure 3, Table 2, and Supplementary Table S6). Of those, seven and three QQIs were identified in the Leu and Ile content datasets. However, no QQI was found in the Val dataset (Table 2 and Supplementary Table S6). The PVE of each QQI was from 2.21% to 5.84% (Table 2). The QQI locus potentially played an important role in the accumulation of the BCAA content. For example, the interaction between QTN-sf0206778257 (G/T) and QTN-sf0427202052 (A/G) underlying the Leu accumulation was observed in QQI-1. Accessions with the highest level of Leu were observed in the combination of the G allele at SNP sf0206778257 and the G allele at SNP sf0427202052, while accessions with the lowest level of Leu were found in the interaction between the G allele at SNP sf0206778257 and the A allele at SNP sf0427202052 (Figure 7A). Additionally, the interaction between QTN-sf0414500497 (G/T) and QTN-sf0810749006 (A/G) associated with the Leu accumulation was identified as the QQI-3 using the 3VmrMLM model. A negative effect on the Leu accumulation of the allele G at SNP sf0414500497 when combined with the allele A at SNP sf0810749006 was observed in this QQI. Accessions carrying the T and G alleles at the two loci showed the highest level of Leu (Figure 7B). In contrast, the interaction between QTN-sf0608765681 (A/G) and QTN-sf0815347583 (G/T) underlying the Leu accumulation was found in QQI-5. Accessions with the highest content of Leu were observed in the combination of the A allele at SNP sf0608765681 and the G allele at SNP sf0815347583, while accessions with the lowest level of Leu were shown in the interaction between the G allele at SNP sf0608765681 and the G allele at SNP sf0815347583 (Figure 7C). However, a different scenario for the interaction between QTN-sf0433008960 (C/T) and QTN-sf1002475086 (C/T) in controlling the Ile content was observed in QQI-8. Accessions with the highest amount of Ile were found in the combination of T and T at the two loci. However, much lower levels of Ile were shown in the accessions with the other three combinations of alleles (Figure 7D).

## 3. Discussion

To elucidate the genetic mechanism underlying the BCAA content in rice, several significant SNPs associated with the BCAA level in leaf and grains were reported [31,32]. In this study, QTN-sf0817467942, QTN-sf0819955092, and QTN-sf0804979333 were identified using the 3VmrMLM model in the Val_BLUP and Val_2013r2 datasets, which were 13.10 kb, 25.26 kb, and 18.29 kb out of the previously reported QTNs (Supplementary Table S1). However, few QTNs underlying the Leu content were reported in previous studies [32]. Among the 3VmrMLM-detected main-effect QTNs associated with the Leu and Val contents, four candidate genes localized in the flanking regions of four reported QTNs/genes were identical with the previous studies related to the BCAA levels in rice (Supplementary Table S1). For instance, the reported genes *OsbZIP18* (*LOC_Os02g10860*) and *OsMCCA* (*LOC_Os12g41250*) in the QTN-sf0205661008 and QTN-sf1225461839 spanned regions were detected in the Leu_2013r2 and Leu_2012r1 content datasets (Supplementary Table S1) [30]. Meanwhile, two candidate genes underlying the Val accumulation (OsMCCB with the gene ID *LOC_Os08g32850* and *OsAUX5* with the gene ID *LOC_Os11g06820*) around the QTN-sf0820240591 and QTN-sf1103421417 were identified in the Val_2012r2 and Val_BLUP datasets (Supplementary Tables S1 and S3) [29,30].

Apart from the reported loci and genes, the new allelic variation of the candidate genes *LOC_Os01g52530* (KEGG annotation: ribose-phosphate pyrophosphokinase in biosynthesis of amino acid pathway) and *LOC_Os06g15420* (KEGG annotation: asparagine synthase in biosynthesis of amino acid pathway) related to the Ile accumulation in grains was uncovered in this study. The biological function of ribose-phosphate pyrophosphokinase and asparagine synthase are confirmed and play important roles in amino acid biogenesis and metabolism. Ribose-phosphate pyrophosphokinase (EC 2.7.6.1) is a key enzyme in catalyzing the ribose-5-phosphate to phosphoribosyl pyrophosphate (5′-phosphoribosyl 1-pyrophosphate, PPRP) [36]. PPRP is an important intermediate in cellular metabolism, which is generally involved in the biosynthesis of amino acid and purine and in the pyrimidine biogenesis and degradation processes [37,38,39]. PPRP also participates in the metabolic link between amino acid and nucleotide as an intermediate metabolite of anthranilate [40]. Asparagine synthetase (EC 6.3.1.1 and EC 6.3.5.4) catalyzes the asparagine synthesis, and asparagine is a precursor of isoleucine in higher plants [41,42,43]. In addition, some transcription factors around the detected main-effect QTNs may become involved in the regulation of the BCAA content variation (Supplementary Table S3). Further validation needs to be carried out in the laboratory to decipher the molecular mechanism of these candidate genes controlling the BCAA accumulation. 

In order to face the challenge of global climate change and meet the food demands of the ever-increasing population, QEI as a crucial role in the interaction between the gene and environment holds the potential to be exploited for the dissection of complex traits in plant GWAS studies. Among the candidate genes of the QEIs related to the three BCAA levels in rice grains, the genetic variation of the LOC_Os03g24460 (KEGG annotation: branched-chain amino acid aminotransferase in amino-acid-related enzyme pathway), LOC_Os01g55590 (KEGG annotation: malonyl-CoA/methylmalonyl-CoA synthetase in valine, leucine, and isoleucine degradation), and LOC_Os12g31820 (KEGG annotation: phosphoserine phosphatase in glycine, serine, and threonine metabolism) genes led to the content alteration of BCAA (Figure 4B, Figure 5B and Figure 6B), which suggests that these genes may contribute to the biological process of BCAA accumulation, which is affected by environmental factors. Branched-chain aminotransferases (BCATs), key enzymes in the interface of the BCAA metabolism, catalyze both the final anabolic step and the first catabolic step in the production of the leucine, isoleucine, and valine in plants [44,45,46,47,48,49]. Particularly, the allelic variation at AtBCAT2 is responsible for the natural variation in the BCAA levels in Arabidopsis seeds [46]. According to the KEGG annotation, the malonyl-CoA/methylmalonyl-CoA synthetase (EC:6.2.1.76 6.2.1.-) participates in the valine, leucine, and isoleucine degradation pathway. BCAAs (valine, leucine, and isoleucine) are structurally related to branched-chain fatty acids [50]. The malonyl-CoA/methylmalonyl-CoA synthetase involved in the BCAA metabolism directly catalyzed the reaction from malonic acid to malonyl-CoA. Malonyl-CoA is a central metabolite that can enhance the lipid content of branched-chain fatty acids in plants and microbes [51,52,53]. In plants, phosphoserine phosphatase is one of three specific enzymes involved in the phosphorylated pathway of serine biosynthesis (PPSB) [54,55]. Notably, the interconversion between serine and threonine is commonly observed in plants. Furthermore, threonine is the precursor of isoleucine synthesis [56]. 

The differences in the metabolic profiles between leaf and grain in rice were reported in a comparative study [32]. Through the GWAS model comparation, our previous study mainly focused on the identification and characterization of main-effect QTLs and genes underlying the valine, leucine, isoleucine, arginine, and tryptophan contents in rice leaf. The 3VmrMLM-detected QEIs related to the above amino acid contents were primarily detected without any natural variation analysis [35]. A total of 23, 16, and 16 QEIs related to valine, leucine, and isoleucine were found. However, the QEIs and QQIs related to the BCAA accumulation in rice grains remain uncovered. In this study, 26, 13, and 14 QEIs related to the BCAA content were identified using the 3VmrMLM model, respectively. Further analyses revealed the natural variations of the three genes controlling the BCAA accumulation in rice grains. Notably, no common QEI was found between the present and previous studies. Compared with the previously proposed model, the technical scheme of 3VmrMLM takes the additive, dominance, additive-by-environment (ae) interaction, and dominance-by-environment (de) interaction effects into account, which were proven to identify the previously reported and novel genes [24]. Moreover, according to the method of the SR4R database, the original genotypic SNP dataset used in the present study was subjected to a linkage disequilibrium (LD) filter to address the redundancy issue of a haplotype block formed by several SNPs within the same LD region. The results shown in this study suggest that using this tag SNP dataset as genotypic data to conduct a GWAS can not only detect the reported genes/loci, but can also help in gaining new findings.

In this study, the additive effect of the BCAA-accumulation-associated QTNs was suggested by the significant correlations between the NFA and BCAA levels (*r* = 0.24–0.66), especially for the Ile content dataset 2012r1 (*r* = 0.66) (Supplementary Figure S3). According to this, the highest Val, Leu, and Ile levels (Val_2013r2, Leu_2013r1, and Ile_2012r1 datasets) were observed in the accessions with four, nine, and fifteen NFAs, such as W127, W155, and W143 (Supplementary Table S7). These suggested that the accessions carrying a few NFAs provide potential targets for the BCAA biofortified rice breeding programs via the loci pyramiding strategy. It was successfully applied in the improvement of various agronomic traits, such as plant height, grain number, hybrid sterility, and disease resistance [57,58,59,60,61]. In the present study, higher BCAA levels were mostly shown in the japonica accessions than in the indica accessions (Figure 1A–C), suggesting that the japonica rice has the potential of the increase in the BACC content in indica rice through the direct hybridization with elite varieties. This was similar with the results reported in our previous study of free amino acid accumulation in rice leaf [35]. In addition, based on a set of QTNs/QTLs with a small effect, the genetic improvement of the yield and disease resistance were successfully facilitated by the application of the genomic selection (GS) breeding approach [62,63,64,65,66,67,68]. Therefore, the small-effect QTNs detected using the 3VmrMLM model might be utilized for the GS breeding in rice with a high BCAA level.

## 4. Materials and Methods

### 4.1. Plant Materials and Sequencing

A diverse panel containing 422 rice accessions of a worldwide rice collection previously released by Huazhong Agricultural University [31] was used for the analyses in this study. Due to the two main subspecies of cultivated rice, accessions belonging to *indica* and *japonica* subspecies were selected for the analyses in this study. Of this genetic panel, 272 accessions are *indica* rice, and 150 accessions are *japonica* rice. According to the geographic information of the 422 accessions, 338 accessions are from Asia, followed by 13 accessions from Europe, 12 accessions from South America, 11 accessions from North America, 5 accessions from Africa, 3 accessions from Oceania, and 40 accessions with unknown status. A randomized complete block design, containing two rows of each accession and ten plants in each row, was applied in the field-grown plants with two replicates in 2012 and 2013, respectively.

For the detection of the genetic variation, each rice accession was sequenced on the Illumina HiSeq 2000 platform to obtain approximately 1 Gb high-quality genome sequences [31]. Using the rice reference genome (version MSU 6.1) and its annotation downloaded from the Rice Genome Annotation Project (http://rice.uga.edu/index.shtml, accessed on 28 June 2023), clean reads were mapped to MSU 6.1 genome using the BWA software (https://sourceforge.net/projects/bio-bwa/, accessed on 28 June 2023) with default settings. After the subsequent process via SAMtools software [69], the SNP joint calling of these 422 rice accessions was conducted using HaplotypeCaller, CombineGVCFs, and GenotypeGVCFs functions with default settings in GATK software (https://gatk.broadinstitute.org/hc/en-us, accessed on 10 August 2023). A set of 3,873,686 high-quality SNPs, obtained using PLINK software (https://zzz.bwh.harvard.edu/plink/, accessed on 28 June 2023) with -maf 0.05 and -geno 0.1 settings, was used for the following analyses in this study. To address the redundancy issue of a haplotype block formed by several SNPs within the same linkage disequilibrium (LD) region, a tag SNP panel including 239,055 SNPs was obtained according to the method proposed by the SR4R database [70]. This tag SNP panel was used as genotypic dataset in the following analyses in the present study.

### 4.2. Metabolite Profiling

For the metabolic profiling, the randomly collected mature grains were pooled from three rice plants as described previously [71]. In brief, four samples (two years by two replicates of each year) for each accession were used for the subsequent metabolomics analyses [32]. To relatively quantify the widely targeted metabolites in dried seed samples, a liquid chromatography–electrospray ionization–tandem mass spectrometry system was employed. Using a mixer mill (MM 400, Retsch GmbH, Haan, Germany) with a zirconia bead for 1.5 min at 30 Hz, 100 mg crushed dried rice grain was extracted overnight at 4 °C with 1.0 mL pure methanol (or 70% aqueous methanol), which contained 0.1 mg/L lidocaine (internal standard) for lipid-soluble metabolites (or water-soluble metabolites). A scheduled multiple reaction monitoring method was used to carry out the quantification of metabolites. By dividing the relative signal intensities of metabolites by the intensities of the internal standard (lidocaine, 0.1 mg/L), the relative intensities of targeted metabolites were normalized. To improve the normality, log2-transformed metabolite data were used for further analysis. A metabolic data matrix with the three relative intensities of free amino acid valine (Val), leucine (Leu), and isoleucine (Ile) from 1688 runs (422 accessions × four sample sets) was generated for the rice genetic panel. The broad-sense heritability *H*^2^ was assessed using the four content datasets of each free amino acid (Val/Leu/Ile). The R package lme4 was used to generate the best linear unbiased prediction (BLUP) datasets for each BCAA (Val, Leu, and Ile) content [72].

### 4.3. Population Stratification and Linkage Disequilibrium Analysis

To dissect the genetic architecture of the diverse panel containing 422 rice accessions, a principal component analysis (PCA) was performed using PLINK software (v1.9) based on the tag SNP dataset obtained above. The concatenated tag SNPs for 422 accessions were used as input data for the phylogenetic analysis. A neighbor-joining (NJ) phylogenetic tree was constructed via the implementation of MEGA-CC software (v11) with the settings of pairwise gap deletion and 1000 bootstrap replicates [73]. The output tree was visualized using the Interactive Tree of Life (iTOL) tool [74]. The population structure was estimated via ADMIXTURE software (v1.3.0) [75]. Using PopLDdecay software, the squared correlation coefficient (*r*^2^) between SNPs was obtained to evaluate the genome-wide LD decay [76]. To estimate the local LD block region in a chromosome, LDBlockShow software (v1.40) was employed [77].

### 4.4. Genome-Wide Association Study

Using the recently proposed 3VmrMLM model [24], the genome-wide association study (GWAS) analyses for 12 Val, Leu, and Ile content datasets were performed on the genetic panel including 422 rice accessions with 239,055 SNPs. The 12 content datasets contained the level of three free amino acids (Val, Leu, and Ile) across two years (2012 and 2013) with two replicates per year. The R package IIIVmrMLM was implemented for the Val-, Leu-, and Ile-content-associated main-effect QTN, QEI, and QQI detection [78]. The parameters for the main-effect QTN detection were method = Single_env, SearchRadius = 20, and svpal = 0.01. The parameters for the QEI detection were method = Multi_env, SearchRadius = 20, and svpal = 0.01. The parameters for the QQI detection were method = Epistasis, SearchRadius = c(0, 1), and svpal = c(0.01, 0.01). The marker trait association (QTN/QEI/QQI) was determined using the threshold of LOD score ≥ 3 [24].

### 4.5. Candidate Gene Prediction and Analysis

To further uncover the genes underlying the Val, Leu, and Ile levels, rice genes localized within the 122 kb (the averaged whole genome LD decay) and local LD block defined flanking regions of each detected QTN/QEI were considered as candidate genes. Kyoto Encyclopedia of Genes and Genomes (KEGG) pathway annotations of candidate genes were analyzed using the KofamKOALA web tool with default settings (https://www.genome.jp/tools/kofamkoala, accessed on 10 August 2023). The functional effect prediction of an SNP on the gene body was obtained from the RiceVarMap database (http://ricevarmap.ncpgr.cn/, accessed on 10 August 2023) and further used for functional allele and content analysis of potential candidate genes through the Wilcoxon non-parametric test at the 5% probability level. Temporal and spatial expression profiles of candidate genes were investigated using the electronic fluorescent pictograph browser (ePlant) web tool (https://bar.utoronto.ca/, accessed on 10 August 2023). The relative contents of each BCAA were obtained according to the method of a comparative metabolic study [79].

## Figures and Tables

**Table 1:** Descriptive statistics of branched-chain amino acid (BCAA) content datasets.

Dataset	Number	Range	Mean	SD	Skewness	Kurtosis	CV (%) ^a^	*H* ^2^
Val-2012r1	422	20.34–24.67	22.28	0.63	0.55	0.77	53.65	0.45
Val-2012r2	422	21.72–25.2	23.26	0.61	0.29	0.02	47.24	
Val-2013r1	422	20.99–27.18	23.61	1.14	0.12	−0.31	92.75	
Val-2013r2	422	20.52–25.11	22.95	0.70	0.07	0.62	54.26	
Leu-2012r1	422	7.02–14.59	10.78	2.09	−0.81	−0.52	99.42	0.49
Leu-2012r2	422	9.85–15.82	12.98	0.91	−0.11	0.09	67.34	
Leu-2013r1	422	7.78–16.13	12.32	1.63	−0.27	−0.34	113.39	
Leu-2013r2	422	9.67–14.72	12.24	0.92	−0.10	−0.05	66.93	
Ile-2012r1	422	7.02–19.97	14.16	2.51	−0.86	1.34	187.57	0.82
Ile-2012r2	422	7.07–20.6	15.93	1.94	−0.35	1.07	156.20	
Ile-2013r1	422	9.06–22.33	15.95	2.13	−0.09	−0.04	219.03	
Ile-2013r2	422	7.02–19.68	15.03	1.91	−0.45	1.12	145.75	

^a^ Calculated from the original dataset. CV: coefficient of variation; SD: standard deviation; *H*^2^: broad-sense heritability. Val, Leu, and Ile represent valine, leucine, and isoleucine. 2012r1, 2012r2, 2013r1, and 2013r2 represent two replicates in 2012 and 2013.

**Table 2:** Detected QTN-by-QTN interactions (QQIs) in grain branched-chain amino acid (BCAA) content datasets. QTN-1 and QTN2 represent the first and second QTNs of each QQI. aa.effect stands for the additive–additive effect.

QQI No.	Trait	QTN-1	QTN-2	aa.effect	LOD	*p*-Value	*R*^2^ (%)
QQI-1	Leu	sf0206778257	sf0427202052	0.25	4.80	2.61 × 10^−6^	4.50
QQI-2	Leu	sf0220165492	sf0600825407	0.22	5.41	6.00 × 10^−7^	4.84
QQI-3	Leu	sf0414500497	sf0810749006	−0.19	5.47	5.14 × 10^−7^	3.90
QQI-4	Leu	sf0606055009	sf1226791763	−0.20	5.19	1.01 × 10^−6^	4.16
QQI-5	Leu	sf0608765681	sf0815347583	0.30	5.02	1.52 × 10^−6^	5.84
QQI-6	Leu	sf0711167346	sf0725292893	0.31	5.61	3.73 × 10^−7^	4.17
QQI-7	Leu	sf1109127718	sf1207700947	−0.29	4.99	1.63 × 10^−6^	3.67
QQI-8	Ile	sf0433008960	sf1002475086	−0.31	6.44	5.15 × 10^−8^	2.31
QQI-9	Ile	sf0622321011	sf0701768847	−0.29	5.79	2.42 × 10^−7^	2.21
QQI-10	Ile	sf0723859952	sf1108192319	0.34	7.40	5.25 × 10^−9^	2.56

## Data Availability

All of the phenotypic and genotypic data used in this study are shared in the Supplementary Materials.

## Supplementary Materials

The following supporting information can be downloaded at https://www.mdpi.com/article/10.3390/plants12162970/s1, Figure S1: KEGG pathway analysis of candidate genes harbored in common QTNs (A) and QEIs (B) associated with valine (Val), leucine (Leu), and isoleucine (Ile) contents. Figure S2: Analyses of the candidate gene LOC_Os01g52530 and LOC_Os06g15420 related to isoleucine (Ile) content. Figure S3: Beeswarm plots illustrating the relationship between the number of favorable alleles (NFA) and valine (Val), leucine (Leu), and isoleucine (Ile) accumulation for the twelve content datasets. Figure S4: Manhattan plots for branched-chain amino acid (BCAA) levels associated QTN-by-environment interactions (QEIs) using 3VmrMLM model. Figure S5: Manhattan plots for branched-chain amino acid (BCAA) levels associated QTN-by-QTN interactions (QQIs) using 3VmrMLM model. QQIs in Val content dataset (A), Leu content dataset (B), and Ile content dataset (C); Table S1: Summary of QTNs detected from branched-chain amino acid (BCAA) content dataset. Table S2: Common QTNs identified from branched-chain amino acid (BCAA) content datasets. Table S3: Candidate genes of common QTNs. Table S4: QTN-by-environment interactions (QEIs) detected from branched-chain amino acid (BCAA) content datasets. Table S5: Candidate genes of QEIs. Table S6: QTN-by-QTN interactions (QQIs) detected from branched-chain amino acid (BCAA) content datasets. Table S7: The number of favorable alleles (NFA) and branched-chain amino acid (BCAA) content of each accession.
